# The Association Between Metabolic Score for Visceral Fat and Cognitive Function Among Older Adults in the United States

**DOI:** 10.3390/nu17020236

**Published:** 2025-01-10

**Authors:** Murong Cheng, Yuchi Meng, Zhenxue Song, Ling Zhang, Yuanjun Zeng, Dongfeng Zhang, Suyun Li

**Affiliations:** Department of Epidemiology and Health Statistics, School of Public Health, Qingdao University, Qingdao 266071, China; chengmurong@qdu.edu.cn (M.C.); mengyuchi@qdu.edu.cn (Y.M.); songzhenxue@qdu.edu.cn (Z.S.); zhangling2@qdu.edu.cn (L.Z.); zengyuanjun@qdu.edu.cn (Y.Z.); zhangdf1961@126.com (D.Z.)

**Keywords:** METS-VF, cognitive function, visceral adipose tissue, NHANES, BMI

## Abstract

Background: Although several studies have demonstrated a link between obesity and cognitive function, the majority have primarily utilized body mass index (BMI) and waist circumference, ignoring the distribution of body fat. Evidence regarding the association of metabolic score for visceral fat (METS-VF), a proposed measurement for visceral adipose tissue (VAT), with cognitive function remains limited. We mainly aimed to investigate this association in older adults in the United States. Methodology: Data were from the National Health and Nutrition Examination Survey (NHANES) 2011 to 2014. Weighted linear regression models were adopted to examine the association of METS-VF and cognitive function scores, with further exploration of these associations across different obesity subgroups. Smoothing curve analysis, along with threshold and saturation effect analysis, were conducted to explore potential non-linear relationships. Results: In the multivariable-adjusted model, participants in the highest quartile (Quartile 4) of METS-VF exhibited a β coefficient of −1.52 [95% CI (−2.43, −0.62)] for the CERAD score compared with those in the lowest quartile (Quartile 1). Threshold and saturation effect analysis revealed non-linear associations of METS-VF with DSST score and Z-score. Conclusions: The findings of this study indicate that elevated METS-VF scores are inversely related to cognitive function, highlighting the importance of considering visceral fat distribution in cognitive health assessments.

## 1. Introduction

With the increasing proportion of the global population aged 65 years and older [1], a range of health issues are likely to arise. Older adults are more susceptible to age-associated memory impairment (AAMI), which can progress to mild cognitive impairment (MCI) and potentially dementia in the absence of any prevention and intervention [2]. It is anticipated that the global population affected by dementia will grow from 57.4 million cases in 2019 to 152.8 million cases by 2050 [3]. There is no established treatment for dementia so far, and the transition process from cognitive decline to dementia is ongoing and irreversible. Thus, it is crucial to explore the modifiable lifestyle factors for MCI to identify and intervene in the population with memory impairment before it progresses to MCI or dementia.

The obesity epidemic has become a significant threat to human health, affecting more than 2 billion individuals worldwide [4]. It has been linked to an increased risk of cardiovascular disease (CVD), mental disorders, and neurodegenerative diseases [5,6,7]. Obese individuals often exhibit reduced hippocampal and cortical volume, impaired performance on different memory tasks, an increased possibility of brain atrophy, and deficits in executive function [8,9,10]. To date, most research examining the relationship between obesity, Alzheimer’s disease (AD), and cognitive decline has focused on body mass index (BMI) and waist circumference (WC) measurements. However, BMI does not accurately reflect body fat distribution, while WC measurements do not distinguish between visceral adipose tissue (VAT) and subcutaneous adipose tissue (SAT) [11,12]. VAT is stored around the abdominal organs, and has been associated with cortical thickness [10], cognitive function [13], and brain volume in older adults [14]. In addition, a significant negative correlation between VAT and total brain volume has been observed in healthy middle-aged individuals, independent of BMI [14]. The metabolic score for visceral fat (METS-VF) was introduced by Bello-Chavolla OY et al. as a surrogate measure of VAT, offering an alternative to more complex instrumental techniques [15]. Several studies have explored the relationships between METS-VF and various diseases [16,17,18]. However, the relationship between METS-VF and cognitive function remains unexplored.

Therefore, our primary objective was to examine the relationship between METS-VF and cognitive function among older adults, further exploring these associations by stratifying participants based on obesity status (measured by BMI) in the United States. Besides that, we would further explore whether threshold effect and saturation effect exist between METS-VF and cognitive function.

## 2. Materials and Methods

### 2.1. Study Population

**Figure 1:**
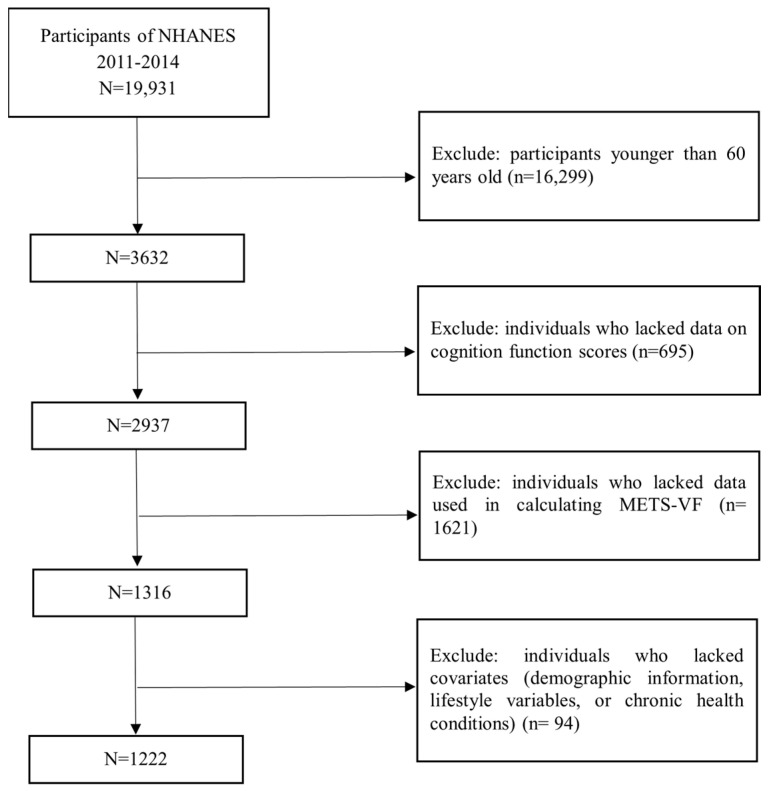
Flow chart of individual inclusion and exclusion.

This research utilized the data from the National Health and Nutrition Examination Survey (NHANES) [19]. Participants were selected using a sophisticated, multistage, stratified random sampling method to ensure the sample reflected the demographics of the U.S. population. Data for our study were obtained from elders who participated in two cycles of the NHANES, spanning the period from 2011–2012 to 2013–2014. Of 3632 individuals aged 60 years or older, participants were excluded based on the following criteria: 695 were removed due to incomplete data on cognitive function scores, 1621 were excluded for missing data necessary to calculate METS-VF, and 94 were excluded due to incomplete data on covariates (demographic details, chronic health issues, or lifestyle factors). Ultimately, 1222 older adults were included in this study (Figure 1).

### 2.2. Assessment of METS-VF and Obesity

METS-VF serves as an index for quantifying visceral adipose tissue (VAT). A high METS-VF value indicates an increased area of VAT. It integrates factors such as age (in years) and gender (male/female), waist-to-height ratio (WHtR) and the metabolic score for insulin resistance index (METS-IR) to provide a comprehensive assessment of visceral fat metabolism, associated health risks, and adipose tissue exposure. Professional technicians performed accurate measurements of BMI, height (HT), and WC at the Mobile Examination Center. Triglycerides (TG) and High-Density Lipoprotein Cholesterol (HDL-C) levels were measured using the Cobas 6000 Chemistry Analyzer, while fasting blood glucose (FBG) was measured with the Roche/Hitachi Cobas C311 Chemistry Analyzer. The units of measurement were as follows: mg/dL for FBG, TG, and HDL-C, and years for age; male was coded as 1 while female was coded as 0. The following formulas were used to calculate METS-VF:

WHtR=WC(cm)/HT(cm)

METS−IR=Ln[(2 × FBG) + TG] × BMI/Ln(HDL−C)

$$METS-VF=4.466+0.011[{\left(Ln(METS-IR)\right)}^{3}]+3.329[{\left(Ln(WHtR)\right)}^{3}]+0.319(gender)+0.594(Ln(age))$$

According to the World Health Organization’s (WHO) guidelines, adults were classified as overweight or obese based on their BMI, which was calculated as weight in kilograms divided by the square of height in meters (kg/m^2^). The classifications were as follows: overweight was defined as a BMI between 25 and 29.9 kg/m^2^, obesity was defined as a BMI of 30 kg/m^2^ or higher, and underweight/normal weight was defined as a BMI below 25 kg/m^2^.

### 2.3. Assessment of Cognitive Function

Cognitive function assessments were conducted on individuals aged 60 years and older in the cycles of NHANES (2011–2014). These assessments were conducted at the Mobile Examination Center (MEC) and included three tests: the Consortium to Establish a Registry for Alzheimer’s disease (CERAD) Word Learning subtest, the Digit Symbol Substitution Test (DSST), and the Animal Fluency Test (AFT). CERAD was used to evaluate participants’ capacity to acquire new language information, both immediately and after a delay, including three consecutive learning tests and a delayed recall test. Participants were instructed to read ten words and immediately recall as many words as possible after a few minutes in each test. CERAD scores were calculated on a scale ranging from 0 to 30 including all three trials for a total score, and from 0 to 10 for the delayed recall score. In AFT, participants were required to name as many animal names as possible within one minute, with one point awarded for each right answer. In our analysis, AFT scores ranged from 3 to 36 and were used to evaluate categorical language fluency, an important aspect of executive function. As a module of the Wechsler Adult Intelligence Test, DSST scores, ranging from 2 to 105 in our analysis, were employed to assess sustained attention, working memory, and processing speed. Subsequently, we calculated the Z-score by averaging the total standardized scores derived from the three cognitive assessments.

### 2.4. Assessment of Covariates

This study included covariates related to key demographic information, chronic health conditions, and lifestyle factors. Categorical variables for demographic and lifestyle factors included gender, race (Non-Hispanic Black, Mexican American, Other Hispanic, Non-Hispanic White, and Other), educational attainment (≤12 years or >12 years), marital status (widowed/divorced/separated, living with a partner, married, and never married), and smoking and drinking status, while age was treated as a continuous variable. Individuals who had smoked fewer than 100 cigarettes in their lifetime were categorized as never smokers, whereas those who had smoked more were classified as smokers. Participants who had consumed fewer than 12 alcoholic drinks in their lifetime were classified as non-drinkers. Those who reported consuming more than 12 alcoholic drinks either within a single year or over their lifetime were further subdivided into two categories: former drinkers and current drinkers, based on their alcohol consumption within the past 12 months [20]. Data regarding participants’ personal medical history, including significant chronic health conditions diagnosed by physicians, such as stroke, diabetes, hypertension, heart disease (encompassing coronary heart disease, congestive heart failure, heart attack, or angina/angina pectoris), and cancer, were obtained through participants’ self-reports.

### 2.5. Statistical Analysis

In our statistical analyses, we followed the CDC analytical guidelines and applied the appropriate sample weights to each participant. This approach was necessary to accurately represent the U.S. population due to the complex multistage cluster survey design of NHANES. Descriptive statistics were utilized, with mean ± standard deviation (SD) or median (interquartile range) used for continuous variables, and frequencies and percentages used for categorical variables. To examine variations between participants exhibiting a different quartile of METS-VF, a chi-square test was performed for categorical variables, while the Kruskal–Wallis test or one-way analysis of variance (ANOVA) were utilized for continuous variables. Weighted linear regression models were then used to calculate β and 95% confidence intervals (CIs) of METS-VF in relation to cognitive function scores. METS-VF was analyzed both as a continuous variable and in quartiles. A linear trend across quartiles was assessed using the median value of each METS-VF quartile. Two models were constructed: a crude model unadjusted for covariate was first examined (Crude Model), followed by an adjusted model adjusted for race, marriage status, education level, smoking, alcohol drinking, and chronic conditions (Adjusted Model). A stratified analysis was conducted to examine whether the association between METS-VF and cognitive function was moderated by obesity status. Weighted linear regression models were used to assess the relationship of METS-VF and cognitive function within each stratified group. Additionally, the relationship between the METS-VF index and cognitive function scores was assessed using generalized additive model (GAM) regression to fit a smoothing curve. When non-linear associations were identified, a log-likelihood ratio test was used to compare two-segment linear regression models with single-linear models, and threshold effects were calculated.

All analyses were performed with R (version 4.2.2), EmpowerStats (version 4.1) and Stata 14.0 (Stata Corporation, College Station, TX, USA) software. A two-sided *p*-value of less than 0.05 was considered statistically significant.

## 3. Results

Table 1 provides the basic information of the 1222 participants included in our analysis, categorized by METS-VF quartile. This included demographic data, chronic health issues, and lifestyle factors. Most baseline characteristics exhibited statistically significant differences across the quartile of METS-VF, with the exceptions of cancer history, marital status, education, and AFT. Participants in the fourth quartile of METS-VF were more likely to be older, male, Non-Hispanic White, smokers, and current drinkers. Additionally, they exhibited higher BMI, more chronic conditions, and lower cognitive function scores compared to those in the first quartile (*p* < 0.05).

Table 2 presents the results from multiple linear regressions models, showing a significant association between higher METS-VF and lower CERAD score. Specifically, in the adjusted model, the CERAD score decreased by 1.52 units for participants in Quartile 4 compared to those in Quartile 1, with a β (95% CI) of −1.52 (−2.43, −0.62). In addition, the CERAD score decreased by 1.18 for each unit increase in METS-VF, while no significant associations were found with other cognitive function scores in the adjusted model.

**Figure 2:**
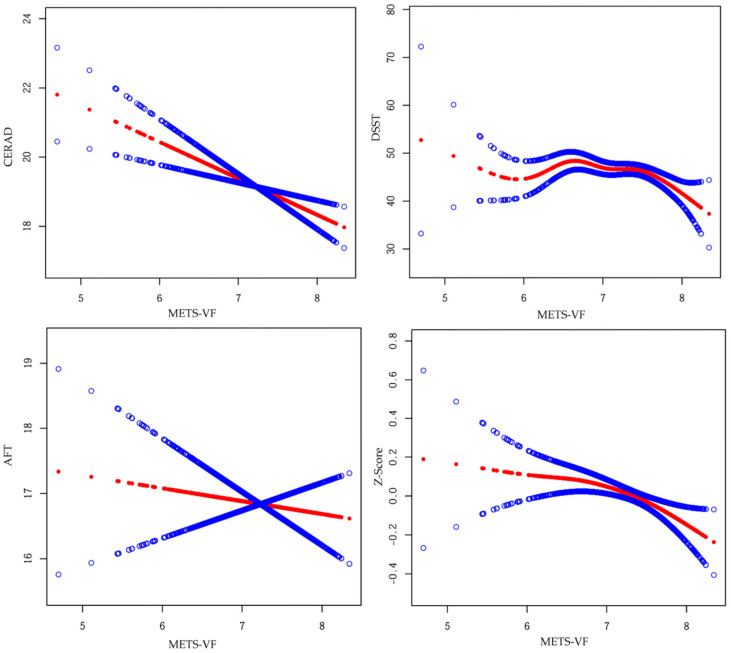
Smooth curve fitting was used to estimate the associations of METS-VF with cognitive function. Abbreviations: METS-VF: metabolic score for visceral fat; CERAD: Consortium to Establish a Registry for Alzheimer’s disease; DSST: Digit Symbol Substitution Test; AFT: Animal Fluency Test. Adjusted for race, marriage status, education level, smoking and drinking status, and chronic conditions (including hypertension, heart disease, cancer, diabetes, and stroke). The middle red line indicates the estimated value, and the blue lines indicate the corresponding 95% confidence intervals.

Figure 2 depicts the smooth curve to estimating the associations between METS-VF and cognitive function. Threshold and saturation effect analyses of METS-VF on cognitive function are presented in Table 3. The DSST score and Z-score demonstrated non-linear associations with METS-VF, with inflection points at k = 7.39 and 7.74, respectively. Notably, when METS-VF reached 7.39, the smooth curve of DSST scores peaked. When METS-VF was below 7.39, the DSST score exhibited a non-significant trend (β = 0.193; 95% CI = −2.17 to 2.56; *p* = 0.873). Conversely, when METS-VF surpassed this threshold, the DSST score decreased by 9.05 points for each unit increase in METS-VF (β = −9.05; 95% CI = −14.01 to −4.09; *p* < 0.001). The Z-score smooth curve reached its peak at a METS-VF of 7.74. When METS-VF was lower than 7.74, the Z-score showed a non-significant downward trend (β = −0.09; 95% CI = −0.18 to 0.01; *p* = 0.073). When METS-VF exceeded the threshold, the Z-score decreased by 0.76 points per unit increase in METS-VF (β = −0.76; 95% CI = −1.31 to −0.21; *p* = 0.007). No non-linear relationship was observed between METS-VF and CERAD or AFT score, as indicated by the log-likelihood ratio test (*p*-value > 0.05).

The findings on the association between each unit increase in METS-VF and cognitive function, stratified by obesity status, are presented in Table 4. In the underweight/normal weight group, the adjusted model indicated that the scores of CERAD, DSST and Z-score decreased by 2.52, 4.92, and 0.36 units, respectively, for each unit increase in METS-VF. In overweight group, the scores of CERAD, DSST, and Z-score decreased by 3.97, 18.6, and 0.73 units, respectively, for each unit increase in METS-VF. In the obesity group, the scores of CERAD, DSST, and Z-score decreased by 3.62, 16.45, and 0.73 units, respectively, for each unit increase in METS-VF. However, no significant associations were observed between AFT and METS-VF across any obesity subgroup in the adjusted models.

## 4. Discussion

This study examined U.S. elders using data from two NHANES cycles (2011–2012 to 2013–2014) to assess the association between METS-VF and cognitive function. Additionally, the study explored these associations in participants stratified by obesity status, as determined by BMI. Furthermore, threshold and saturation effect analyses were conducted to evaluate the presence of non-linear relationships and to identify the optimal METS-VF level for cognitive function protection. The findings indicated that higher METS-VF levels was associated with lower CERAD scores, independent of demographic variables, chronic health conditions, and lifestyle factors. No significant associations were observed with other cognitive function scores. However, threshold and saturation effect analyses revealed non-linear associations between METS-VF and both DSST score and Z-score. This may account for the lack of significance in the linear regression results. Differently from the results based on all participants, significant negative correlations between METS-VF and both DSST score and Z-score were observed among participants stratified by obesity.

In this study, participants in the fourth quartile of METS-VF had a 1.52 unit decrease in CERAD score compared to those in the first quartile. In addition, the smooth curve and threshold and saturation effect analysis revealed non-linear associations between METS-VF and both DSST score and Z-score, with inflection points at k = 7.39 and 7.74, respectively. Obesity causes increases in adipose tissue (AT), including VAT and SAT, while METS-VF has been proposed as a simple alternative to complex instrumental measurements for assessing VAT. Several studies have suggested that VAT may impair cognitive function [10,21].

The mechanisms underlying the association between VAT and cognitive function remain poorly understood. One potential mechanism may involve metabolic or systemic inflammation. The adipose tissue is an endocrine organ, capable of secreting inflammatory cytokines, such as interleukin-6 (IL-6), and tumor necrosis factor-α, which are associated with an increased risk of dementia [22]. Besides that, an elevated level of inflammatory cytokines could further lead to deep white matter hyperintensity, a known risk factor for dementia [23,24]. Furthermore, the secretion of adipokines, such as leptin and adiponectin [25], by adipose tissue may also influence cognitive function through their neuroprotective effects [25,26]. However, in the context of obesity, despite the increased production of leptin, the blood–brain barrier (BBB) permeability of leptin is reduced due to the onset of leptin resistance [27], while the production of adiponectin is reduced [25]. Therefore, in the case of excessive abdominal obesity, the protective effects of adipokines on cognitive decline may be weakened. Additionally, the association between visceral fat and reduced brain volume may also represent a potential mechanism [10]. Central obesity is more strongly correlated with brain volume than overall obesity, with visceral fat being a particularly prominent and robust component of abdominal obesity [14], and the reduction in brain volume is linked to decreased cognitive performance [28].

This study offers several advantages. First of all, by utilizing a nationally representative sample of the U.S. population, this research ensures that the findings are generalizable to older adults across the United States. Furthermore, this analysis adjusted for key demographic variables, chronic health conditions, and lifestyle factors to isolate their associations with the outcomes. However, the study is subject to certain limitations. Firstly, the cross-sectional design limits the ability to establish causal relationships. Further prospective studies are warranted to confirm this association in the future. Moreover, the reliance on self-reported questionnaire data may introduce recall bias. In addition, the NHANES database does not include serum inflammatory cytokines, such as TNF-α and IL-6 levels. Consequently, we are unable to investigate the role of inflammatory factors on the association between cognition function and METS-VF. Finally, since the study was conducted within the U.S. with a predominantly white sample, the generalizability of the findings to other ethnic groups may be limited.

## 5. Conclusions

In conclusion, the findings of this research indicated that elevated METS-VF scores were inversely related to cognitive function scores. Moreover, this association was particularly evident among participants stratified by obesity. These results suggested that cognitive decline prevention might be effectively achieved through screening, monitoring, and controlling METS-VF.

## Figures and Tables

**Table 1:** Basic characteristics of included sample by quartile of METS-VF (n = 1222).

Characteristics	All Participants	Quartile of METS-VF				*p*-Value
		Q1 (5.44–6.95)	Q2 (6.95–7.30)	Q3 (7.30–7.57)	Q4 (7.57–8.22)	
No. of participants	1222	306	305	306	305	
Age, median (IQR) ^c^	69 (63, 76)	66 (63, 72)	68 (63, 75)	69 (64, 76)	70 (65, 77)	<0.001
Gender, n (%) ^b^						
Male	603 (49.35)	94 (30.72)	128 (41.97)	161 (52.61)	220 (72.13)	<0.001
Female	619 (50.65)	212 (69.28)	177 (58.03)	145 (47.39)	85 (27.87)	
Race, n (%) ^b^						
Mexican American	108 (8.84)	20 (6.54)	22 (7.21)	31 (10.13)	35 (11.48)	<0.001
Non-Hispanic White	619 (50.65)	151 (49.35)	155 (50.82)	142 (46.41)	171 (56.07)	
Non-Hispanic Black	245 (20.05)	59 (19.28)	68 (22.30)	63 (20.59)	55 (18.03)	
Other Hispanic	128 (10.47)	22 (7.19)	34 (11.15)	42 (13.73)	30 (9.84)	
Other	122 (9.98)	54 (17.65)	26 (8.52)	28 (9.15)	14 (4.59)	
Marital status, n (%) ^b^						
Married	726 (59.41)	176 (57.52)	188 (61.64)	180 (58.82)	182 (59.67)	0.674
Widowed/Divorced/Separated	388 (31.75)	94 (30.72)	90 (29.51)	104 (33.99)	100 (32.79)	
Never married	70 (5.73)	24 (7.84)	16 (5.25)	15 (4.90)	15 (4.92)	
Living with a partner	38 (3.11)	12 (3.92)	11 (3.61)	7 (2.29)	8 (2.62)	
BMI, mean (SD) ^a^	28.99 (6.13)	23.14 (2.72)	26.93 (2.76)	30.22 (3.71)	35.71 (6.04)	<0.001
Education, n (%) ^b^						
≤12 years	305 (24.96)	61 (19.93)	73 (23.93)	84 (27.45)	87 (28.52)	0.061
>12 years	917 (75.04)	245 (80.07)	232 (76.07)	222 (72.55)	218 (71.48)	
Smoking status, n (%) ^b^						
Non-Smokers	605 (49.51)	173 (56.54)	149 (48.85)	157 (51.31)	126 (41.31)	0.002
Smokers	617 (50.49)	133 (43.46)	156 (51.15)	149 (48.69)	179 (58.69)	
Alcohol drinking, n (%) ^b^						
Non-Drinkers	193 (15.79)	52 (16.99)	50 (16.39)	52 (16.99)	39 (12.79)	0.002
Former Drinkers	320 (26.19)	53 (17.32)	84 (27.54)	85 (27.78)	98 (32.13)	
Current Drinkers	709 (58.02)	201 (65.69)	171 (56.07)	169 (55.23)	168 (55.08)	
Hypertension, n (%) ^b^	754 (61.70)	144 (47.06)	178 (58.36)	213 (69.61)	219 (71.08)	<0.001
Stroke, n (%) ^b^	83 (6.79)	16 (5.23)	29 (9.51)	12 (3.92)	26 (8.52)	0.017
Heart attack, n (%) ^b^	226 (18.49)	33 (10.78)	42 (13.77)	59 (19.28)	93 (30.16)	<0.001
Diabetes, n (%) ^b^	282 (23.08)	30 (9.80)	54 (17.70)	83 (27.12)	115 (37.70)	<0.001
Cancer, n (%) ^b^	245 (20.05)	63 (20.59)	65 (21.31)	51 (16.67)	66 (21.64)	0.388
CERAD, mean (SD) ^a^	19.14 (4.54)	20.17 (4.58)	19.13 (4.39)	19.03 (4.58)	18.23 (4.34)	<0.001
AFT, mean (SD) ^a^	16.84 (5.45)	17.31 (5.53)	17.11 (5.73)	16.16 (5.07)	16.76 (5.41)	0.06
DSST, mean (SD) ^a^	46.14 (17.27)	50.51 (17.56)	46.35 (17.43)	45.32 (16.91)	42.35 (16.23)	<0.001

Abbreviations: METS-VF: metabolic score for visceral fat; CERAD: Consortium to Establish a Registry for Alzheimer’s disease; DSST: Digit Symbol Substitution Test; AFT: Animal Fluency Test; BMI: body mass index (kg/m^2^). ^a^ *p*-value was tested by one-way analysis of variance (ANOVA); ^b^ *p*-value was tested by Chi-square test; ^c^ *p*-value was tested by Kruskal–Wallis test.

**Table 2:** Associations of METS-VF with cognitive function: NHANES: 2011–2014.

	CERAD	*p*	DSST	*p*	AFT	*p*	Z-Score	*p*
	β (95% CI)		β (95% CI)		β (95% CI)		β (95% CI)	
**Crude model ^a^**								
Per 1-Unit Increase in METS-VF	**−1.51 (−2.28, −0.73)**	**<0.001**	**−4.97 (−8.37, −1.58)**	**0.005**	**−0.98 (−1.96, 0.002)**	**0.050**	**−0.27 (−0.42, −0.11)**	**0.001**
Quartile of METS-VF								
Q1 (5.44–6.95)	1 (reference)		1 (reference)		1 (reference)		1 (reference)	
Q2 (6.95–7.30)	**−1.55 (−2.59, −0.50)**	**0.005**	−4.02 (−8.29, 0.25)	0.064	−0.69 (−1.98, 0.60)	0.282	**−0.23 (−0.44, −0.02)**	**0.030**
Q3 (7.30–7.57)	**−1.14 (−1.98, −0.31)**	**0.009**	**−4.77 (−8.25, −1.29)**	**0.009**	**−1.76 (−2.99, −0.52)**	**0.007**	**−0.28 (−0.45, −0.12)**	**0.001**
Q4 (7.57–8.22)	**−2.13 (−3.12, −1.14)**	**<0.001**	**−8.09 (−12.89, −3.28)**	**0.002**	−1.03 (−2.39, 0.33)	0.133	**−0.38 (−0.59, −0.16)**	**0.001**
*p* trend		**<0.001**		**0.001**		**0.049**		**0.001**
**Adjusted model ^b^**								
Per 1-Unit Increase in METS-VF	**−1.18 (−1.90, −0.47)**	**0.002**	−1.84 (−5.04, 1.36)	0.249	−0.32 (−1.27, 0.63)	0.495	−0.14 (−0.29, 0.005)	0.058
Quartile of METS-VF								
Q1 (5.44–6.95)	1 (reference)		1 (reference)		1 (reference)		1 (reference)	
Q2 (6.95–7.30)	**−1.36 (−2.33, −0.39)**	**0.007**	−1.91 (−5.32, 1.50)	0.262	−0.24 (−1.43, 0.95)	0.684	−0.15 (−0.33, 0.03)	0.094
Q3 (7.30–7.57)	−0.80 (−1.61, 0.002)	0.051	−1.86 (−5.16, 1.44)	0.259	−1.03 (−2.30, 0.23)	0.105	−0.16 (−0.32, 0.001)	0.052
Q4 (7.57–8.22)	**−1.52 (−2.43, −0.62)**	**0.002**	−3.50 (−7.89, 0.89)	0.114	−0.05 (−1.33, 1.22)	0.935	−0.18 (−0.37, 0.008)	0.060
*p* trend		**<0.001**		**<0.001**		0.123		**<0.001**

Abbreviations: METS-VF: metabolic score for visceral fat; CERAD: Consortium to Establish a Registry for Alzheimer’s disease; DSST: Digit Symbol Substitution Test; AFT: Animal Fluency Test. ^a^ unadjusted; ^b^ adjusted for race, marriage status, education level, smoking and drinking status, and chronic conditions (including hypertension, heart disease, cancer, diabetes, and stroke). Bold font indicates that the result is statistically significant.

**Table 3:** Threshold effect and saturation effect analysis of METS-VF on cognitive function.

METS-VF	CERAD		DSST		AFT		Z-Score	
	β (95% CI)	*p*-Value	β (95% CI)	*p*-Value	β (95% CI)	*p*-Value	β (95% CI)	*p*-Value
Standard multivariable linear regression model	−1.05 (−1.59, −0.52)	<0.001	−2.17 (−3.92, −0.42)	0.015	−0.20 (−0.82, 0.43)	0.534	−0.13 (−0.22, −0.05)	0.002
Inflection point (k)	7.68		7.39		7.80		7.74	
Less than k	−0.84 (−1.45, −0.23)	0.007	0.193 (−2.17, 2.56)	0.873	−0.01 (−0.68, 0.66)	0.976	−0.09 (−0.18, 0.01)	0.073
More than k	−3.17 (−6.06, −0.27)	0.032	−9.05 (−14.01, −4.09)	<0.001	−3.87 (−8.90, 1.16)	0.131	−0.76 (−1.31, −0.21)	0.007
*p* for log-likelihood ratio test		0.142		**0.003**		0.146		**0.022**

Abbreviations: METS-VF: metabolic score for visceral fat; CERAD: Consortium to Establish a Registry for Alzheimer’s disease; DSST: Digit Symbol Substitution Test; AFT: Animal Fluency Test. Adjusted for race, marriage status, education level, smoking and drinking status, and chronic conditions (including hypertension, heart disease, cancer, diabetes, and stroke). Bold font indicates that the result is statistically significant.

**Table 4:** Stratified analysis of associations between per unit increase in METS-VF and cognitive function.

Subgroups	Underweight/Normal Weight		Overweight		Obesity	
	β (95% CI)	*p*-Value	β (95% CI)	*p*-Value	β (95% CI)	*p*-Value
**CERAD**	**−2.52 (−3.98, −1.06)**	**0.001**	**−3.97 (−5.35, −2.58)**	**<0.001**	**−3.62 (−5.43, −1.80)**	**<0.001**
**DSST**	**−4.92 (−8.81, −1.03)**	**0.015**	**−18.60 (−24.09, −13.11)**	**<0.001**	**−16.45 (−26.66, −8.23)**	**<0.001**
**AFT**	−1.35 (−2.84, 0.14)	0.075	−1.37 (−3.88, 1.14)	0.275	−2.47 (−5.15, 0.20)	0.069
**Z-score**	**−0.36 (−0.59, −0.13)**	**0.003**	**−0.73 (−1.01, −0.46)**	**<0.001**	**−0.73 (−1.05, −0.42)**	**<0.001**

Abbreviations: METS-VF: metabolic score for visceral fat; CERAD: Consortium to Establish a Registry for Alzheimer’s disease; DSST: Digit Symbol Substitution Test; AFT: Animal Fluency Test. Adjusted for race, marital status, education level, smoking and drinking status, and chronic conditions (including hypertension, diabetes, heart disease, stroke, and cancer). Bold font indicates that the result is statistically significant.

## Data Availability

The data are available at https://wwwn.cdc.gov/nchs/nhanes/default.aspx (accessed on 7 January 2025).

## References

[1] World Population Policies|Population Division. https://www.un.org/development/desa/pd/data/world-population-policies.

[2] Vega J.N., Newhouse P.A. (2014). Mild Cognitive Impairment: Diagnosis, Longitudinal Course, and Emerging Treatments. Curr. Psychiatry Rep..

[3] (2022). GBD 2019 Dementia Forecasting Collaborators Estimation of the Global Prevalence of Dementia in 2019 and Forecasted Prevalence in 2050: An Analysis for the Global Burden of Disease Study 2019. Lancet Public Health.

[4] Corona G., Rastrelli G., Filippi S., Vignozzi L., Mannucci E., Maggi M. (2014). Erectile Dysfunction and Central Obesity: An Italian Perspective. Asian J. Androl..

[5] Elias M.F., Elias P.K., Sullivan L.M., Wolf P.A., D’Agostino R.B. (2005). Obesity, Diabetes and Cognitive Deficit: The Framingham Heart Study. Neurobiol. Aging.

[6] Li X., Shi X., Tan Y., Yu Y., Tang C., Xu G., Zhang X., Liao H., Mai X., Chen W. (2022). Metabolic Indexes of Obesity in Patients with Common Mental Disorders in Stable Stage. BMC Psychiatry.

[7] Więckowska-Gacek A., Mietelska-Porowska A., Wydrych M., Wojda U. (2021). Western Diet as a Trigger of Alzheimer’s Disease: From Metabolic Syndrome and Systemic Inflammation to Neuroinflammation and Neurodegeneration. Ageing Res. Rev..

[8] Coppin G., Nolan-Poupart S., Jones-Gotman M., Small D.M. (2014). Working Memory and Reward Association Learning Impairments in Obesity. Neuropsychologia.

[9] Dye L., Boyle N.B., Champ C., Lawton C. (2017). The Relationship between Obesity and Cognitive Health and Decline. Proc. Nutr. Soc..

[10] Isaac V., Sim S., Zheng H., Zagorodnov V., Tai E.S., Chee M. (2011). Adverse Associations between Visceral Adiposity, Brain Structure, and Cognitive Performance in Healthy Elderly. Front. Aging Neurosci..

[11] Nuttall F.Q. (2015). Body Mass Index: Obesity, BMI, and Health: A Critical Review. Nutr. Today.

[12] Poirier P., Després J.-P. (2003). Waist Circumference, Visceral Obesity, and Cardiovascular Risk. J. Cardiopulm. Rehabil..

[13] Anand S.S., Friedrich M.G., Lee D.S., Awadalla P., Després J.P., Desai D., de Souza R.J., Dummer T., Parraga G., Larose E. (2022). Evaluation of Adiposity and Cognitive Function in Adults. JAMA Netw. Open.

[14] Debette S., Beiser A., Hoffmann U., Decarli C., O’Donnell C.J., Massaro J.M., Au R., Himali J.J., Wolf P.A., Fox C.S. (2010). Visceral Fat Is Associated with Lower Brain Volume in Healthy Middle-Aged Adults. Ann. Neurol..

[15] Bello-Chavolla O.Y., Antonio-Villa N.E., Vargas-Vázquez A., Viveros-Ruiz T.L., Almeda-Valdes P., Gomez-Velasco D., Mehta R., Elias-López D., Cruz-Bautista I., Roldán-Valadez E. (2020). Metabolic Score for Visceral Fat (METS-VF), a Novel Estimator of Intra-Abdominal Fat Content and Cardio-Metabolic Health. Clin. Nutr..

[16] Liu H., Dong H., Zhou Y., Jin M., Hao H., Yuan Y., Jia H. (2024). The Association between Metabolic Score for Visceral Fat and Depression in Overweight or Obese Individuals: Evidence from NHANES. Front. Endocrinol..

[17] Zhu Y., Zou H., Guo Y., Luo P., Meng X., Li D., Xiang Y., Mao B., Pan L., Kan R. (2023). Associations between Metabolic Score for Visceral Fat and the Risk of Cardiovascular Disease and All-Cause Mortality among Populations with Different Glucose Tolerance Statuses. Diabetes Res. Clin. Pract..

[18] Wu W., Pei Y., Wang J., Liang Q., Chen W. (2024). Association between Visceral Lipid Accumulation Indicators and Gallstones: A Cross-Sectional Study Based on NHANES 2017–2020. Lipids Health Dis..

[19] NHANES Questionnaires, Datasets, and Related Documentation. https://wwwn.cdc.gov/nchs/nhanes/default.aspx.

[20] Cheung C.-L., Sahni S., Cheung B.M.Y., Sing C.-W., Wong I.C.K. (2015). Vitamin K Intake and Mortality in People with Chronic Kidney Disease from NHANES III. Clin. Nutr..

[21] Schwartz D.H., Leonard G., Perron M., Richer L., Syme C., Veillette S., Pausova Z., Paus T. (2013). Visceral Fat Is Associated with Lower Executive Functioning in Adolescents. Int. J. Obes..

[22] Darweesh S.K.L., Wolters F.J., Ikram M.A., de Wolf F., Bos D., Hofman A. (2018). Inflammatory Markers and the Risk of Dementia and Alzheimer’s Disease: A Meta-Analysis. Alzheimer’s Dement..

[23] Association between Visceral Fat and Brain Structural Changes or Cognitive Function. https://www.mdpi.com/2076-3425/11/8/1036.

[24] Lampe L., Zhang R., Beyer F., Huhn S., Kharabian Masouleh S., Preusser S., Bazin P.-L., Schroeter M.L., Villringer A., Witte A.V. (2019). Visceral Obesity Relates to Deep White Matter Hyperintensities via Inflammation. Ann. Neurol..

[25] Forny-Germano L., De Felice F.G., Vieira M.N.d.N. (2018). The Role of Leptin and Adiponectin in Obesity-Associated Cognitive Decline and Alzheimer’s Disease. Front. Neurosci..

[26] Kim J.Y., Barua S., Jeong Y.J., Lee J.E. (2020). Adiponectin: The Potential Regulator and Therapeutic Target of Obesity and Alzheimer’s Disease. Int. J. Mol. Sci..

[27] Gruzdeva O., Borodkina D., Uchasova E., Dyleva Y., Barbarash O. (2019). Leptin Resistance: Underlying Mechanisms and Diagnosis. Diabetes Metab. Syndr. Obes..

[28] Hackert V.H., den Heijer T., Oudkerk M., Koudstaal P.J., Hofman A., Breteler M.M.B. (2002). Hippocampal Head Size Associated with Verbal Memory Performance in Nondemented Elderly. Neuroimage.

